# Alkylated MXene–Carbon Nanotube/Microfiber Composite Material with Flexible, Superhydrophobic, and Sensing Properties

**DOI:** 10.3390/ma17184499

**Published:** 2024-09-13

**Authors:** Siyu Wang, Dawei Xia, Xinyu Xu, Haoyang Song, Yongquan Qing

**Affiliations:** 1 Shenyang Fire Science and Technology Research Institute of MEM, Shenyang 110034, China; wsy19870722@163.com (S.W.); fstime@163.com (D.X.); 2 School of Materials Science and Engineering, Northeastern University, Shenyang 110819, China; shy15031513253@163.com

**Keywords:** superhydrophobic, robustness, sensing, anti-fouling

## Abstract

Superhydrophobic strain sensors are highly promising for human motion and health monitoring in wet environments. However, the introduction of superhydrophobicity inevitably alters the mechanical and conductive properties of these sensors, affecting sensing performance and limiting behavior monitoring. Here, we developed an alkylated MXene–carbon nanotube/microfiber composite material (AMNCM) that is simultaneously flexible, superhydrophobic, and senses properties. Comprising a commercially available fabric substrate that is coated with a functional network of alkylated MXene/multi-walled carbon nanotubes and epoxy–silicone oligomers, the AMNCM offers high mechanical and chemical robustness, maintaining high conductivity and strain sensing properties. Furthermore, the AMNCM strain sensor achieves a gauge factor of up to 51.68 within a strain range of 80–100%, and exhibits rapid response times (125 ms) and long-term stability under cyclic stretching, while also displaying superior direct/indirect anti-fouling capabilities. These properties position the AMNCM as a promising candidate for next-generation wearable devices designed for advanced environmental interactions and human activity monitoring.

## 1. Introduction

With the development and widespread application of wearable technologies, the demand for wearable strain sensors has been continuously increasing [1,2]. Wearable strain sensors, which monitor biomechanical signals such as human motion and deformation, are primarily utilized in fitness monitoring, medical diagnosis, smart homes, and other domains [3,4,5]. However, traditional wearable strain sensors still face numerous challenges in terms of wear comfort and durability, particularly when exposed to environments influenced by water, sweat, microorganisms, acids, alkalis, and salts. One major vulnerability stems from the sensor’s susceptibility to changes in humidity, which can cause a sharp decline in sensing performance [6]. This vulnerability arises mainly from the formation of a water film on the sensor surface, which disrupts the sensor and destabilizes its structure, ultimately shortening its service life [7,8]. In practical applications, exposure to aqueous environments is inevitable for these sensors. The immersion of conductive sensors in water can lead to short circuits in the conductive pathways, potentially damaging the sensor components and posing safety risks to users [9,10]. Moreover, the infiltration of water molecules can cause the conductive materials to decompose, detach, or oxidize, resulting in the decreased electrical conductivity of the sensing components [11,12].

Inspired by natural phenomena, such as the surfaces of lotus and peanut leaves that easily allow water droplets to roll off, superhydrophobic surfaces (with a water CA greater than 150° and an SA less than 10°) have been successfully applied in various fields, including self-cleaning [13], anticorrosion [14], wearable sensors [12,15,16], antifreezing [17], and oil–water separation [18]. Currently, the coupling design of flexible strain sensing materials with superhydrophobic surfaces has emerged as the most advanced direction in the development of sensing materials. This design strategy enables traditional flexible wearable strain sensing materials to stably participate in human daily activities in an unencapsulated form, facilitating long-term stability in monitoring human behavior in complex environments. For example, Ding et al. [19] fabricated thermoplastic elastomer/carbon nanotube nanocomposite sheets with good conductivity and superhydrophobicity, demonstrating high strain sensing and sensitivity for monitoring human behavior in humid environments. Jia et al. [20] developed a superhydrophobic fabric-based strain sensing material that achieved high sensitivity (with a sensing coefficient of 169) under small strains of 0–10%, effectively monitoring minute human motions and physiological signals even in harsh conditions. Ma et al. [21] reported a long-lived fluorinated carbon nanodot-line/microfiber coupling textile that had simultaneously excellent stretchability, water-repellency, and conductivity. 

Despite the remarkable technological strides made in recent years, the pursuit of achieving optimal mechanical and conductive properties while integrating superhydrophobic surfaces into strain sensing materials remains a formidable challenge. The surface modification process inevitably impacts the inherent mechanical and electrical properties of the sensors, potentially compromising their overall sensing performance [22,23,24]. Furthermore, the current state-of-the-art materials still fall short in fully embracing the multifunctional requirements of modern wearable applications, underscoring the need for continuous research and development efforts to bridge these gaps and strike an ideal balance between high-performance sensing capabilities and robust environmental resilience [25]. Therefore, the development of strain sensing materials that harmoniously blends exceptional sensing indicators with superhydrophobic stability continues to pose a significant challenge for researchers in this field.

In this work, we developed a superhydrophobic and robust alkylated MXene–carbon nanotube/microfiber composite material (AMNCM) composed of a commercially available, cost-effective fabric substrate and a functional filler network (MXene/MWCNTs). The process involved immersing the fabric in a nano-filler solution followed by a subsequent coating to encapsulate and protect the fibers. This treatment enhanced the surface roughness via MXene and MWCNTs while a fluorine-free alkylation process lowered the surface energy, offering environmental friendliness and imparting superhydrophobic properties. Furthermore, the AMNCM exhibited exceptional serviceability and strain sensitivity, and it demonstrated waterproof and self-cleaning capabilities compared to other conductive fabric sensors, making it a promising candidate for an integrated wearable device capable of monitoring human activities both in air and underwater.

## 2. Experimental Section

### 2.1. Materials

Lithium fluoride (CAS 7789-24-4, Shanghai, China) was procured from Aladdin Biochemical Technology Co., Ltd. Titanium carbide aluminum (CAS 12537-81-4, Beijing, China) powder was supplied by Beijing Forsman Technology Co., Ltd. Multi-walled carbon nanotubes (MWCNTs, CAS 308068-56-6, Jiangsu, China) were purchased from Jiangsu Xianfeng Nano Material Co., Ltd. Octadecyltrichlorosilane (OTS, CAS 112-04-9, Shanghai, China) was purchased from Shanghai McLean Biochemical Technology Company. All chemicals were analytical grade reagents and were used as received.

### 2.2. Preparation of the Alkylated MXene–Carbon Nanotube

Lithium fluoride (5 g) was dissolved in hydrochloric acid (9 M) and magnetically stirred for 1 h. Then, Ti_3_AlC_2_ powder (5 g) was added, and the mixture was magnetically stirred at 35 °C for 24 h to form a stable suspension. The suspension was washed, centrifuged until it was a nearly neutral pH, redissolved in deionized water, and centrifuged again to collect concentrated Ti_3_C_2_T_x_ nanosheet supernatants until transparent. The final precipitate was vacuum-dried, weighed to determine the MXene concentration, and diluted to synthesize an MXene colloidal solution (5 mg·mL^−1^).

MXene (0.2 g) and OTS (0.08 mL) were combined in an absolute ethanol solution (40 mL) and magnetically stirred at 45 °C for 2 h to form the alkylated MXene (OTS–MXene). Meanwhile, multi-walled carbon nanotubes (0.12 g) were dispersed in absolute ethanol, with OTS (0.05 mL) added, and the mixture was stirred magnetically at 45 °C for 2 h to produce alkylated MWCNTs (OTS–MWCNTs). Subsequently, our as-prepared epoxy-based silicone oligomers [26] were incrementally introduced into the suspension containing both alkylated MXene and MWCNTs. The resulting mixture was sonicated for 15 min at room temperature and then magnetically stirred for another 2 h, resulting in the formation of an alkylated MXene–carbon nanotube suspension.

### 2.3. Preparation of the Alkylated MXene–Carbon Nanotube/Microfiber Composite Material 

The fabric was precisely cut, soaked in sodium hydroxide (0.01 M) for 30 min, and then thoroughly rinsed with deionized water. Afterward, it was oven-dried at 80 °C to create hydroxylated fibers. The fibers were subsequently immersed in a uniform suspension of alkylated MXene–carbon nanotubes, dried under vacuum at a consistent temperature, and this dipping and drying process was repeated five times to ensure a thorough and even coating. Finally, a uniform nano-medium was sprayed onto the fiber surfaces and dried to form an AMNCM.

### 2.4. Characterization

The contact angles (CAs) and sliding angles (SAs) were examined by an optical contact angle meter device (JCY-2, Fangrui, Shanghai, China) with 4–6 μL water droplets. The microstructures of the material surfaces were observed and analyzed by field-emission scanning electron microscopy (SEM, Apreo 2C, Thermo Fisher, Waltham, MA, USA) at an accelerating voltage of 10 kV with a laser confocal microscope (LSCM, OLS3100, Tokyo, Japan). Surface chemical compositions were measured by Fourier transform infrared spectroscopy (FTIR, K-Alpha, Berlin, Germany) using a standard KBr pellet method in the range from 4000 to 500 cm^−1^ and X-ray photoelectron spectroscopy (XPS, K-Alpha, San Francisco, CA, USA) using a monochromatic Al_Kα_ (hv = 1486.6 eV) radiation source. The AMNCM was cut to a uniform size of 4 × 1 cm, with its textile ends attached to copper wires using conductive silver paste. The electrodes were then sealed with waterproof tape to shield them from water damage. This assembled AMNCM strain sensor was subsequently employed in strain sensing tests and human motion detection. The resistance variation of the AMNCM was recorded using a resistance collector (Fluke 2638A, Houston, TX, USA). The AMNCM was stretched using a micro-force material testing machine (Tytron250, Mechanical Testing & Simulation, Detroit, MI, USA) with one end fixed and the other end linearly elongated at a constant speed of 10 mm/min.

## 3. Results and Discussion

### 3.1. Design for Producing the AMNCM

**Figure 1:**
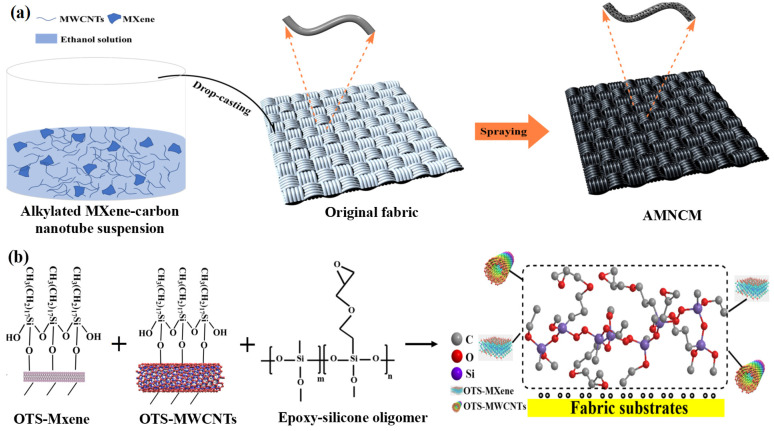
(**a**) Design strategies for AMNCM. (**b**) Reaction mechanism illustration for the formation of the AMNCM.

The AMNCM was composed of a low-surface-energy nano-media filling phase (reinforcing media) and a textile fiber matrix (Figure 1a). The fabric fibers served as the primary load-bearing component, determining the mechanical properties such as the tensile strength, resilience, and bending strength of the material. The nano-filling media endowed the material with hydrophobic, wear-resistant, and stress-balancing properties, bonding with the textile fiber matrix to form an integrated whole and achieving a composite enhancement of performance. Generally, composite materials are composed of two or more multi-phase substances with different physical and chemical properties. In such materials, the nano-media act as the dispersed phase, surrounded and dispersed by the continuous phase, with an interface between the two phases. The stability of the composite material is closely related to the microstructure, properties, and bonding strength of the interface. To enhance the robustness of the AMNCM, two criteria must be followed: (i) concerning the formation of chemical bonds between the continuous and dispersed phases, we selected surface-hydroxylated textile fibers as the continuous phase and chemically modified MXene and MWCNTs as the dispersed phase, with interfacial bonding enhancing the bonding strength; (ii) the nano-media contain specific adhesive molecules and superhydrophobic substances. The epoxy–silicone oligomer used in the experiment exhibited high adhesiveness and non-toxic properties, and the combination of OTS–MWCNTs and OTS–MXene further enhanced the material’s strength and controlled its wettability.

In the composite material system of the AMNCM, the epoxy–silicone oligomer served as a binder, which promoted the formation of micro–nano rough structures on the surface of the MXene and MWCNTs and compensated for the poor adhesion and susceptibility to detachment in OTS–MXene/OTS–MWCNTs (Figure 1b). This was primarily attributed to the epoxy–silicone oligomer connecting the OTS–MXene and OTS–MWCNTs with the textile fiber matrix through functional group reactions. Its functional groups bonded with the oxygen atoms of the matrix to form ester bonds and formed coordination bonds with MXene, enhancing the adhesion and internal bonding strength of the nano-media. Additionally, the epoxy–silicone oligomer leveraged its surface adherence to intersperse between the matrix and nano-media, facilitating the formation of an interconnected network structure upon curing. This interconnectedness strengthened the bond between the dispersed and continuous phases, ultimately enhancing the material’s overall stability.

### 3.2. Microstructure and Composition

**Figure 2:**
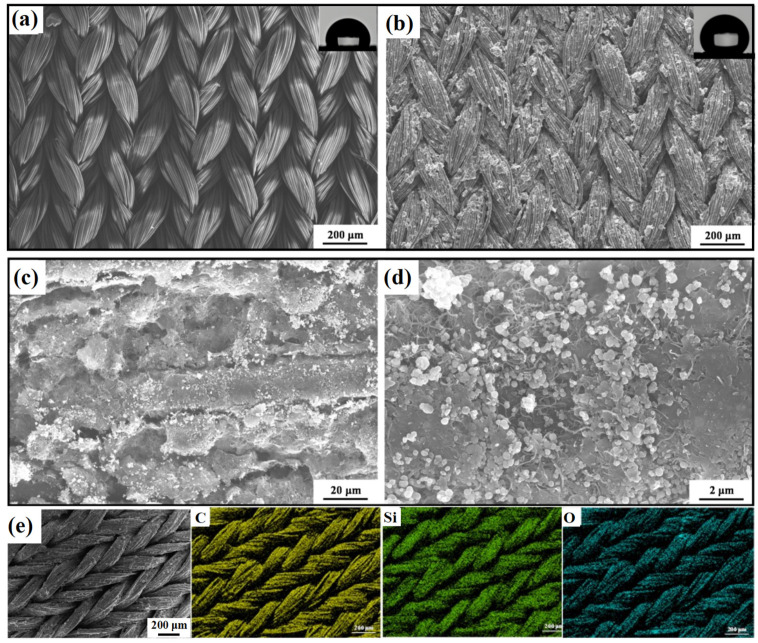
SEM images of (**a**) original fabric and (**b**–**d**) AMNCM surface at different magnifications. (**e**) Elemental mappings of main elements C, O, Si, and Cl on the AMNCM surface.

The SEM images in Figure 2 show the microtopography of fabric fibers and the AMNCM surface under different magnifications. The fabric surface exhibited a superhydrophilic state with a water contact angle of 72.3° (Figure 2a). This surface was woven by multiple fiber filaments, which following hydroxylation treatment, exhibited abundant hydroxyl groups that predominantly contributed to its superhydrophilic properties. In contrast, the AMNCM surface showed a remarkable superhydrophobic state with a water contact angle of 158.6° (Figure 2b). Further observation revealed a rough surface with numerous regular micro/nano structures, which were formed by the accumulation of OTS–MXene nanosheets, adhesives, and OTS–MWCNTs, with pore spacing ranging from ~1 to 3 μm. Air became entrapped within these pores, forming an extremely thin air layer (Figure 2c,d). This tight aggregation of nanomaterials formed a nuanced micro/nano multi-level roughness, essential for achieving exceptional superhydrophobic properties. Furthermore, the EDS elemental mapping in Figure 2e reveals that the constituent elements of the AMNCM surface, with atomic percentages of Si at 6.72%, C at 75.51%, and O at 14.39%, were uniformly distributed, ensuring that the hydrophobic nanoparticles, comprising these elements, homogeneously covered the fabric fibers without local agglomeration.

**Figure 3:**
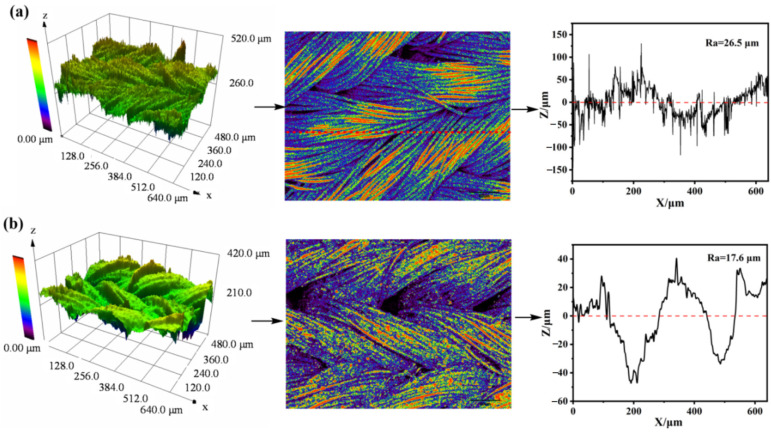
LSCM topographical images of (**a**) original fabric and (**b**) AMNCM surface.

Figure 3a,b reveal that both the fabric and AMNCM surfaces exhibited comparable three-dimensional topologies, characterized by surface roughness (Ra) values of 26.5 μm and 17.6 μm, respectively. This similarity underscored the fundamental influence of the substrate on the rough, irregular profiles, as visually validated by SEM imaging. The incorporation of nano–media into the AMNCM surface led to the formation of a consolidated, refined structural matrix, resulting in a reduction in surface roughness. This refinement facilitated the rapid establishment of an air cushion layer, which was crucial for effective droplet repulsion or rebound. The even distribution of nano-media both on and within the substrate acted to homogenize the surface, eliminating voids and irregularities for a smoother texture. This smoother surface enhanced the fabric’s ability to entrap air, creating a durable anti-adhesion barrier that inhibited droplet attachment and promoted their swift detachment upon contact. Consequently, the AMNCM surface exhibited significantly improved waterproofing and anti-wetting properties, marking a substantial advancement in the resistance to droplet adherence compared to untreated fabrics.

**Figure 4:**
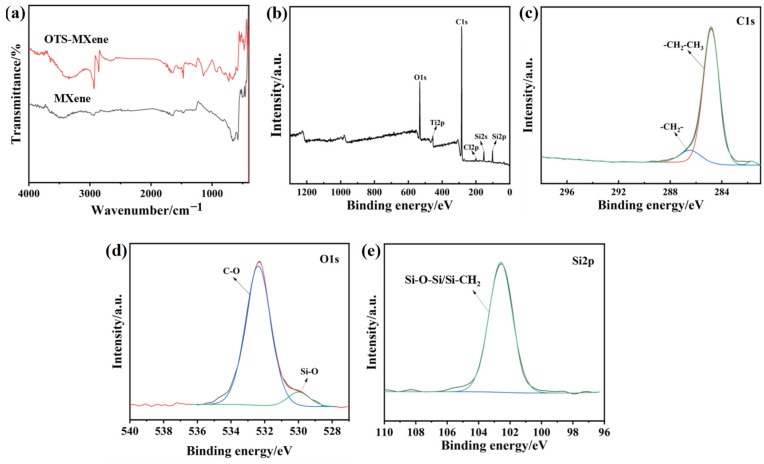
(**a**) FTIR spectra of OTS–MXene and MXene. (**b**) XPS survey spectra, (**c**) C1s, (**d**) O1s, and (**e**) Si2p high-resolution XPS spectra of the AMNCM surface.

To explore the underlying reasons for the hydrophobic properties exhibited by AMNCM, we conducted a thorough analysis of the chemical composition on its surface using FTIR and XPS. Figure 4a shows a comparison of FTIR spectra for MXene before and after modification with OTS. Notably, the modified MXene spectrum exhibited two new prominent absorption peaks within the 2800–3000 cm^−1^ range, corresponding to the –CH_2_– and –CH_3_ functional groups, indicating the successful grafting of low-surface-energy groups onto the MXene surface. Figure 4b shows the survey spectra of the AMNCM surface, revealing strong signals for C1s, O1s, Si2s, and Si2p, along with a weaker signal for Ti2p. The C1s high-resolution spectrum in Figure 4c comprised two peaks at 283.6 eV and 284.1 eV, attributed to carbon atoms in C–C and C–H bonds, respectively, originating from OTS and the epoxy–silicone oligomer. The O1s high-resolution spectrum in Figure 4d can be deconvoluted into two peaks at 532.7 eV and 529.8 eV, corresponding to C–O and Si–O, respectively. In the Si2p high-resolution spectrum (Figure 4e), new peaks that arose at 102.6 eV corresponding to Si–O–Si/Si–CH_2_ were detected. These further confirmed the successful grafting and assembly of OTS and the epoxy–silicone oligomer onto the MXene/MWCNTs surface, providing the necessary low-surface-energy substances a foundation for the superhydrophobic sensor. Moreover, the epoxy–silicone oligomer served as an adhesive, ensuring strong adhesion between the nano-medium and fabric fibers.

### 3.3. Mechanical and Chemical Robustness

**Figure 5:**
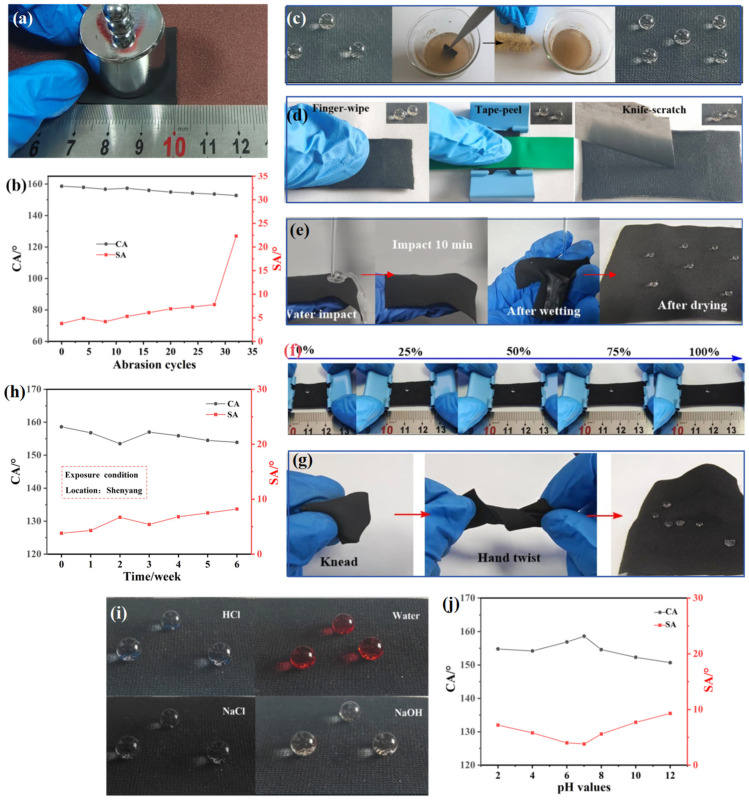
(**a**) Schematic illustration of sandpaper abrasion test experimental setup. (**b**) Change in the contact angle and sliding angle with mechanical abrasion lengths for AMNCM. (**c**) Image of AMNCM wettability before and after sewage washing. (**d**) Image of the AMNCM wettability after finger wiping, tape peeling, and knife scratching. Image of wettability of the AMNCM after (**e**) water impact, (**f**) stretching, and (**g**) bending kink. (**h**) Effect of outdoor exposure time on the stability of the AMNCM. (**i**) The wetting state of water, acid, alkali, and salt on the AMNCM surface. (**j**) CA and SA of droplets with different pH values on the AMNCM surface.

The mechanical robustness of the AMNCM, exemplified by its resilience to stress and deformation without damage, was underscored by rigorous tests involving abrasion, scratching, and stretching. This characteristic, pivotal to wettability, was initially addressed [27,28]. Linear abrasion tests on sandpaper were conducted for the AMNCM, with water CAs and SAs measured before and after abrasion. As shown in Figure 5a, a 25 mm × 25 mm AMNCM surface was placed on 800-grit sandpaper and moved 10 cm along a ruler in two perpendicular directions under a pressure of 1.6 kPa (a 100 g load), defined as one abrasion cycle. Figure 5b shows the impact of abrasion cycles on the CAs and SAs of the AMNCM surface. The results indicated that the AMNCM surface maintained good superhydrophobicity with a water CA above 152.7° after less than 28 abrasion cycles; however, further abrasion cycles hindered water droplets from rolling off the surface. To further evaluate the mechanical robustness of the AMNCM surface, it was immersed in a muddy water mixture and then brushed eight times with an experimental brush dipped in the mixture. Such a surface maintained a CA greater than 150° and an SA less than 10° (Figure 5c). In addition, the material underwent blade scratching, finger wiping, and tape peeling tests to simulate external environmental impacts during practical applications (Figure 5d). The results demonstrated that after multiple blade scratches, finger wipes, and tape peelings, water droplets remained spherical on the surface and easily rolled off at a tilt angle less than 10°, indicating the material’s resistance to extreme and harsh conditions.

Next, the AMNCM was subjected to a water flow impact experiment under a running tap. As shown in Figure 5e, even after enduring a 10 min water flow impact until fully wetted, the material still exhibited superhydrophobic properties upon drying, indicating its good resistance to external impact forces. To investigate the influence of microcracks generated by dynamic stretching on the material’s hydrophobic stability, dynamic tensile tests were conducted. These results showed that in the original state and under 25%, 50%, 75%, and 100% stretching conditions, the droplets on the AMNCM surface remained spherical, with contact angles above 150° (Figure 5f), indicating that microcracks produced by dynamic stretching did not affect its hydrophobic stability. Furthermore, after being kneaded and twisted, the AMNCM surface still maintained excellent hydrophobicity, with droplets appearing spherical (Figure 5g), further demonstrating the material’s good tolerance to deformations under external forces.

Chemical robustness ensures the long-term stability of the AMNCM’s properties, safeguarding against chemical degradation. Given that the hydrophobic properties of the AMNCM may be compromised when used outdoors due to exposure to impurities, dust, and corrosive substances, the material was first placed in an outdoor environment to monitor changes in its surface water CA and SA over time (Figure 5h). The results indicated that after being exposed outdoors for six weeks, the material’s CA remained almost unaffected, with only minor changes in the SA, and the CA consistently stayed above 153°, indicating that the material’s superhydrophobic characteristics remained stable under long-term outdoor exposure. Subsequently, to verify the applicability of the AMNCM in acid–base and salt environments (such as acid rain), the effects of droplets with different pH values on its surface hydrophobicity were investigated. Figure 5i shows that droplets with pH values of 2 (light blue HCl), 7 (red water and colorless NaCl), and 12 (pale yellow NaOH) all formed spherical shapes on the AMNCM surface. Figure 5j shows the CAs and SAs of water droplets with varying pH values on the AMNCM surface. Observations from the figure indicate that across a pH range from 2 to 12, the CAs on the material surface consistently exceeded 150°, while the SAs were all less than 10°, indicating that the material exhibited excellent resistance to acid, alkali, and salt.

### 3.4. Anti-Fouling Performance

**Figure 6:**
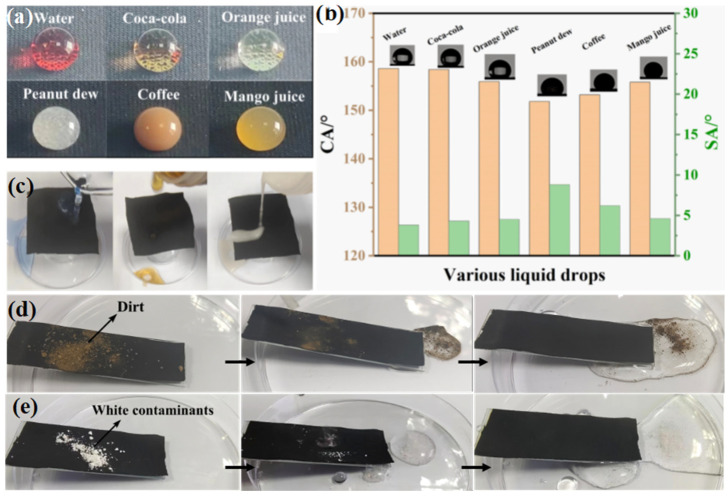
(**a**) Different types of liquid droplets with spherical shapes on coating surface, namely red-dyed water, Coca-Cola, orange juice, peanut dew, coffee, and mango juice. (**b**) The CAs and SAs of different droplets on the AMNCM surface. (**c**) Photo of acid, Coca-Cola, and milk being poured onto the AMNCM surface. Self-cleaning ability of the AMNCM against (**d**) soil and (**e**) white contaminants.

Anti-fouling capability is a critical indicator for evaluating the performance of AMNCMs in practical applications [29,30]. Depending on the surface decontamination mechanism, anti-fouling capability can be classified into direct or indirect anti-fouling abilities. As shown in Figure 6a, regardless of variations in color, viscosity, and the composition of common aqueous liquids encountered in daily life (such as water, Coca-Cola, orange juice, peanut dew, coffee, and mango juice), the AMNCM surface remained uncontaminated. These droplets easily rolled off its surface without leaving any trace, with all droplets exhibiting CAs greater than 150° and SAs less than 10° (Figure 6b), indicating exceptional super-repellency against various aqueous liquids. To assess the direct anti-fouling ability of the AMNCM surface, three different aqueous liquids were poured onto it. During pouring, transparent liquids such as acid, Coca-Cola, and milk, easily rolled off the horizontally placed AMNCM surface without leaving any residue, indicating the material’s outstanding direct anti-fouling ability (Figure 6c).

To further elucidate the indirect anti-fouling ability (i.e., self-cleaning ability) of the AMNCM surface, hydrophilic contaminants such as soil or white powder with particle sizes (D) larger than the pore size (P) of the wettable material surface were employed. Figure 6d and e show that water droplets moving across the AMNCM surface could effortlessly remove dirt and other contaminants, restoring its cleanliness, indicating a weak interaction between the hydrophilic contaminants and the hydrophobic surface of the material. These phenomena align with the “lotus effect” and are mainly ascribed to the micro-/nano-scale protrusion structures on the AMNCM surface, which entrapped air to create an air cushion. Upon droplet contact, the Cassie state was maintained, preventing surface contamination. The remarkable direct and indirect anti-fouling capabilities of the AMNCM ensure its long-term industrial applicability.

### 3.5. Sensing Performance

**Figure 7:**
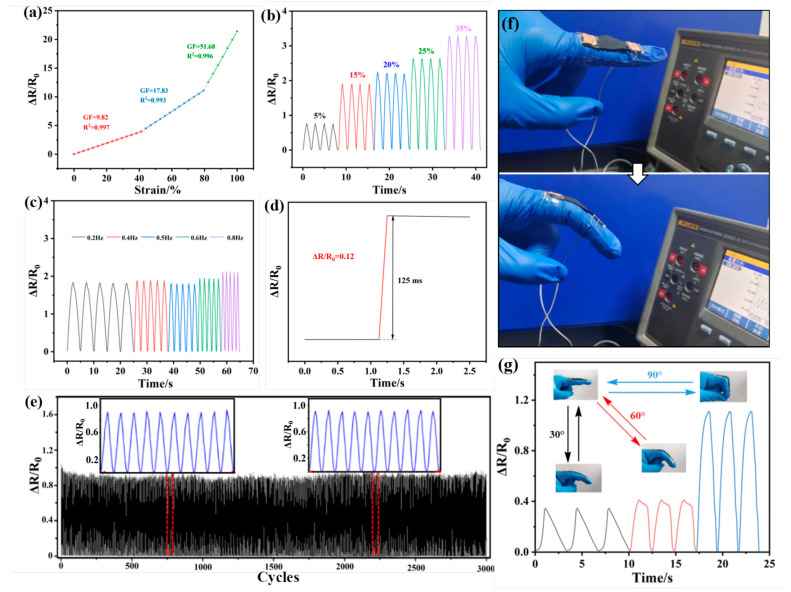
(**a**) Resistance response of the AMNCM strain sensor at different strain regions. (**b**) Relative resistance variation of the AMNCM strain sensor under various stretching/releasing conditions. (**c**) Periodic strain sensing behavior of the AMNCM strain sensor with different frequencies. (**d**) Response time of the AMNCM strain sensor. (**e**) Long-term strain sensing performance of the AMNCM strain sensor under 3000 stretching and releasing cycles (20% strain). Human behavior monitoring map of the AMNCM strain sensor for (**f**) finger bending changes and (**g**) resistance changes at different bending angles.

During cyclic tension–release processes, the relative change in surface resistance (ΔR/R_0_, where R_0_ is the initial resistance and ΔR is the real-time resistance variation) of the AMNCM was monitored using a Fluke device to assess the sensitivity, strain range, and stability of the designed strain sensor. Tensile experiments yielded typical strain–stress curves and detailed mechanical properties of the superhydrophobic sensor, with the gauge factor (GF, calculated as (ΔR/R_0_)/ε, where ε is the sensor strain) serving as a key parameter indicating sensor sensitivity. Resistance changes during stretching were recorded, revealing the functional relationship between ΔR/R_0_ and strain. According to the GF calculation, the slope of the curve represents the actual sensitivity. Sample sensitivity varied across different strain regions, with GF gradually increasing as the composite strain rose, averaging 9.82, 17.83, and 51.68 within the ε ranges of 0–40%, 40–80%, and 80–100%, respectively, as shown in Figure 7a. This high sensitivity is attributed to the layered structure of the AMNCM, which amplified strain effects while maintaining good conductivity. When the composite fabric was mounted on a tensile testing machine, the resistance increased with fabric extension. During this process, the spacing between the nano-media on the material surface continuously expanded, yet the circuit remained connected, demonstrating the fabric’s dynamic stability under large deformations. 

To further evaluate the real-time response capability of the AMNCM strain sensor, multiple tension/release tests were conducted within a strain range of 5% to 35%, as shown in Figure 7b. Even at a low strain of 5%, the multi-cycle electrical signal curves remained stable, demonstrating good reproducibility and confirming the sensor’s low detection limit. To confirm the sensor’s frequency-independent sensing characteristics in practical applications, cyclic tension/release tests were performed on the AMNCM within a 0–15% strain range at increasing frequencies of 0.2, 0.4, 0.5, 0.6, and 0.8 Hz. The peak resistance values stayed remarkably stable as the frequency increased, indicating the composite material’s potential for monitoring human activities or mechanical movements at diverse speeds (Figure 7c). The AMNCM strain sensor exhibited a brief instant response with a response time of 125 ms at a strain of 100%, as shown in Figure 7d. This indicates that the composite material could respond to strain changes within 125 ms, demonstrating its rapid response speed, which is beneficial for applications requiring real-time monitoring such as human motion detection, health monitoring, and security domains. 

To ensure long service life in practical applications, strain sensors must maintain their sensing properties without significant fatigue failure. To investigate the long-term stability of AMNCM strain sensors, 3000 cycles of 0 to 20% strain were applied at a rate of 480 mm/min (Figure 7e). The stable conductive network and layered structure conducive to releasing external stress ensured the stability of the cyclic curves. By comparing the resistance response curves of 10 randomly selected cycles before and after the 3000 cycles (inset in Figure 7e), no significant changes in the shape and peak values of the response curves were observed, indicating the composite material’s good dynamic durability. Further morphological observations of the composite were conducted to explore the sensing mechanism. Initially, MXene and MWCNTs intertwined to form a conductive network and layered structure. Upon stretching, minute cracks emerged, allowing for the detection of resistance changes. As stretching intensified, the cracks widened, leading to an increase in ΔR values. With further strain, the substrate fabric stretched, causing damage to the conductive network, an increase in resistance, and a rise in the GF value. The incorporation of the epoxy–silicone oligomer reinforcement conferred high stability to the sensor, preventing the complete disruption of the conductive network even under extreme stretching. The recovery of fibers led to the disappearance of cracks and the restoration of the conductive network, demonstrating exceptional cyclic stability.

Considering the sensor’s flexibility, superhydrophobicity, durability, high sensitivity, and rapid real-time dynamic response, a strain sensor device was constructed using copper wires and a Fluke, enabling the direct connection to the human body. As shown in Figure 7f, the AMNCM exhibited outstanding performance in human motion monitoring, highlighting its potential for applications in smart medical diagnostic systems. Notably, the high sensitivity of the sensor allowed it to detect large joint movements. For example, when the strain sensor was attached to the finger of a tester, bending and relaxing the finger caused the sensor to deform, eliciting a resistive response. This response increased and decreased periodically with joint motion, forming a wavy ΔR/R_0_ curve, indicating the real-time monitoring capability of the AMNCM strain sensor. Furthermore, the detection curves for each cycle exhibited high consistency, indicating excellent repeatability, while varying degrees of finger bending (30°, 60°, and 90°) were distinctly discernible, attributable to different extents of conductive path disruptions caused by varying levels of deformation, which resulted in unique ΔR/R_0_ responses (Figure 7g).

## 4. Conclusions

In summary, we successfully developed an AMNCM, which featured a coating of a commercial fabric substrate with a functional filler network comprised of alkylated MXene/MWCNTs and an epoxy–silicone oligomer, endowing the material with flexible, superhydrophobic, and sensing properties. Notably, the AMNCM consistently maintained its excellent superhydrophobicity even under harsh mechanical abrasions, dynamic stretching, and exposure to corrosive environments. Its layered structure enhanced strain sensitivity, achieving a gauge factor of up to 51.68 with rapid response times of 125 ms, facilitating accurate and timely monitoring. Moreover, the long-term stability of the AMNCM under 3000 cycles of stretching and releasing confirmed its potential for practical applications. In human behavior monitoring tests, the AMNCM strain sensor demonstrated the ability to detect even minute finger bending motions. The AMNCM uniquely combined durability, high sensitivity, and environmental resistance, positioning it as a promising candidate for next-generation wearable devices for advanced monitoring. Further efforts should focus on exploring diverse functionalization strategies for MXene and MWCNTs to tailor conductivity, flexibility, and hydrophobicity to AMNCM’s intended applications.

## Data Availability

All data generated or analyzed during this study are included in the present article.
